# Transoral Approach to the Craniovertebral Junction: A Case Series of Three Rare Pathologies

**DOI:** 10.7759/cureus.94046

**Published:** 2025-10-07

**Authors:** Morteza Sadeh, Hadeel Mansour, Javed Iqbal, Gabriel Gonzales-Portillo, John Souter, Sergey Neckrysh, Nicholas Callahan, Gursant Atwal

**Affiliations:** 1 Neurosurgery, University of Illinois at Chicago, Chicago, USA; 2 Oral and Maxillofacial Surgery, University of Illinois at Chicago, Chicago, USA

**Keywords:** anterior spinal artery aneurysm, craniovertebral junction, cvj anomalies, klippel-feil syndrome, transoral approach, ventral cervicomedullary pial avm

## Abstract

The transoral approach (TOA) to the craniovertebral junction (CVJ) provides direct midline access to ventral compressive pathologies but remains associated with notable technical challenges and complications. We present our institution’s case series of rare CVJ pathologies, highlighting operative nuances and patient outcomes.

Three extremely rare and complex CVJ cases, including Klippel-Feil syndrome with severe stenosis, anterior spinal artery aneurysm, and ventral cervicomedullary pial arteriovenous malformation, undergoing tailored transoral interventions were retrospectively analyzed for technical details, outcomes, and complications. Successful ventral decompression was achieved in all cases. Unique operative techniques, including transmandibular and transpalatal osteotomies, were critical for accessing these rare lesions. Complications, including cerebrospinal fluid leakage, prolonged dysphagia, and staged posterior fusion, underscored the surgical complexity. At the six-month follow-up, all patients showed neurological stability or improvement.

The TOA remains an essential technique for the management of ventral CVJ pathology, particularly in cases where posterior or endonasal approaches are insufficient. Optimal outcomes are achieved through detailed preoperative planning, multidisciplinary collaboration, and vigilant management of approach-related complications. This series illustrates the ongoing role of TOA in the management of rare and technically demanding CVJ pathologies where alternative methods may prove inadequate*.*

## Introduction

The craniovertebral junction (CVJ), also referred to as the craniocervical junction (CCJ), is a complex anatomical region that connects the skull base to the upper cervical spine [1,2]. The CVJ houses critical neural and vascular structures, including the brainstem, vertebral arteries, lower cranial nerves, and cervical spinal cord. This makes it particularly vulnerable to injuries and compressive pathologies such as neoplastic lesions, basilar invagination, atlantoaxial dislocation (AAD), rheumatoid arthritis, and odontoid fractures [3-8]. These conditions may arise from congenital anomalies, degenerative changes, or trauma. Owing to their deep-seated and anterior location adjacent to major neurovascular structures, CVJ lesions present significant surgical challenges. If left untreated, they can result in severe neurological deficits, often necessitating surgical intervention [9-12].

The transoral approach (TOA) provides a direct midline corridor to the CVJ through the oral cavity, enabling surgeons to access and decompress ventral lesions while minimizing the need for extensive soft tissue and muscle dissection [13-18]. First described by Albert Kanavel in 1919 and later refined and popularized in the 1970s and 1980s by Arnold H. Menezes, the TOA became established as the gold standard for managing complex midline CVJ pathologies [2,19].

Over the past century, the TOA has undergone substantial refinements, evolving into several variations tailored to the specific location and nature of CVJ lesions [2]. Although alternative strategies such as the endoscopic endonasal approach (EEA), posterior approaches, and lateral approaches have gained traction, the TOA continues to be a preferred option in select cases requiring direct midline exposure [19].

Here, we present our institution’s case series of rare CVJ pathologies requiring transoral access, with emphasis on anatomical considerations, technical nuances, complications, and postoperative management.

## Case presentation

Case 1: Klippel-Feil syndrome (transmandibular transoral approach)

**Figure 1:**
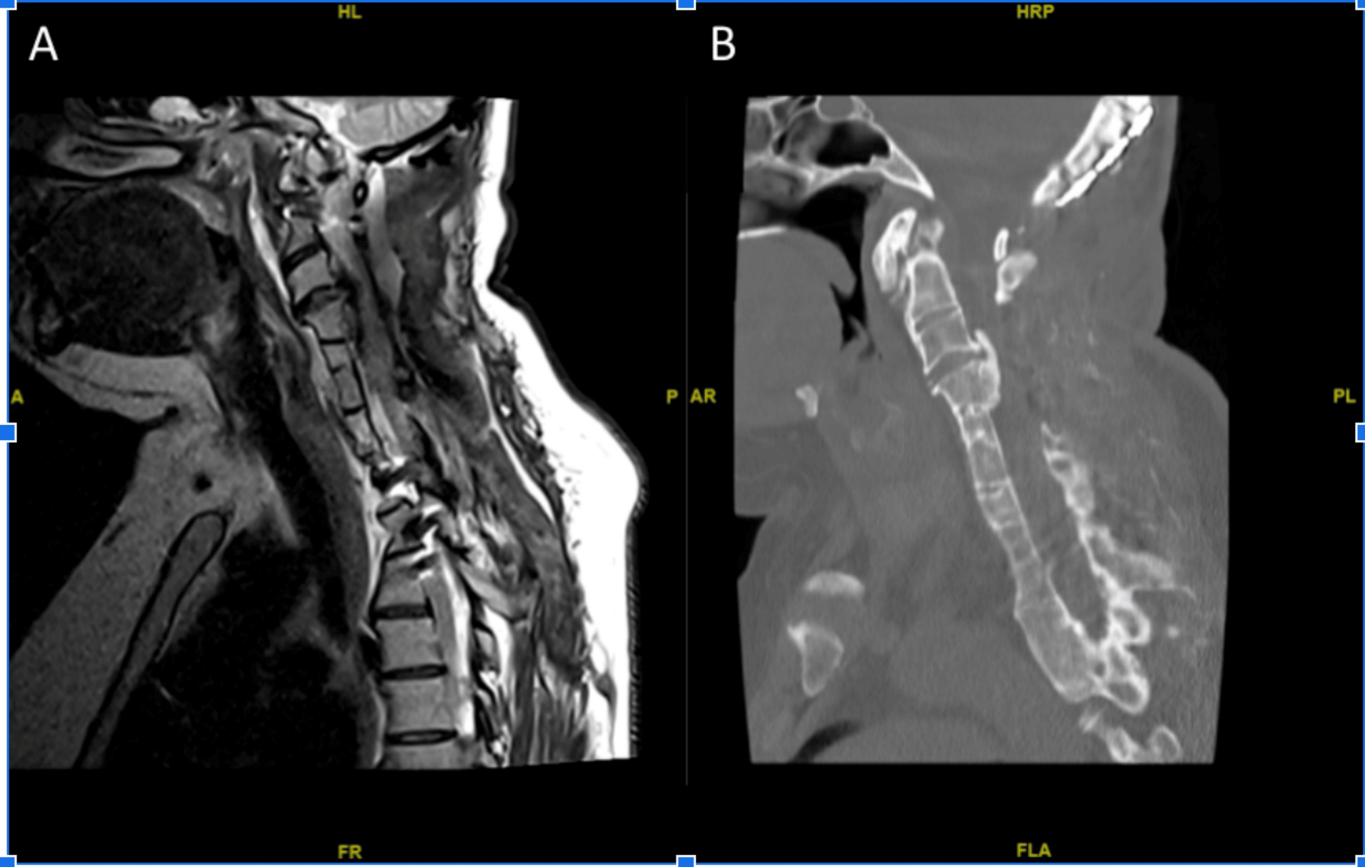
(A) Sagittal T2-weighted MRI view showing severe ventral spinal cord compression at the level of C3. (B) Sagittal CT scan demonstrating congenital segmentation anomalies consistent with Klippel-Feil syndrome, including fusion of cervical vertebrae, hypoplasia of the clivus, and a cranially positioned odontoid process.

The patient was a 38-year-old woman with Klippel-Feil syndrome, presenting with progressive weakness and myelopathy. There was severe canal stenosis and cord compression, most pronounced at the C3 level (Figure 1). The patient initially underwent C3-6 cervical laminectomies, which resulted in some improvement in her symptoms. However, her symptoms continued to decline, including worsening deltoid and hand grip weakness and gait imbalance, despite maximal physical therapy. A cervical corpectomy and posterior fusion were planned, but the anterior approach was aborted due to difficulties with a safe approach to the anterior cervical region, secondary to the patient’s short and rigid neck structure. The patient’s neurological function continued to decline postoperatively, despite a vigorous physical therapy program, due to persistent stenosis.

Additionally, the high-riding hyoid bone, retropositioned mandible, and severely limited cervical extension restricted the surgical corridor and visualization of the upper cervical vertebral bodies, making standard anterior and transoral routes inadequate for safe decompression. A transmandibular approach was recommended as a last resort. A thorough discussion was conducted with the patient and her family to educate them about the potential morbidities associated with this approach, including the need for alternative nutrition, tracheostomy creation, and a high likelihood of cerebrospinal fluid (CSF) leak requiring diversion procedures.

Operation

**Figure 2:**
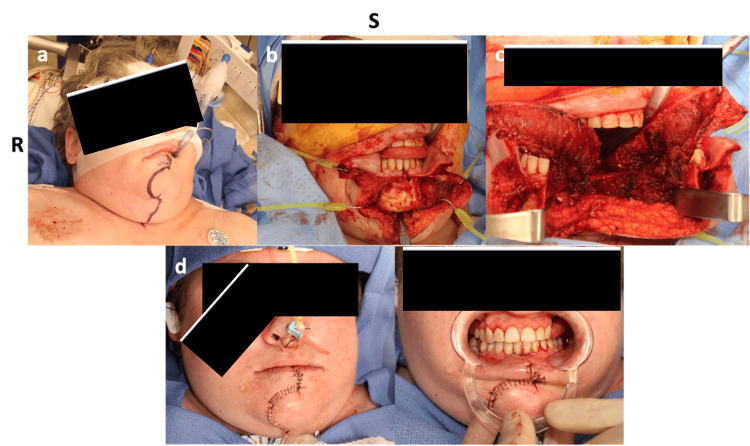
Panel (a) shows preoperative incision planning for midline mandibulotomy. Panel (b) demonstrates intraoperative exposure following mandibular osteotomy and soft tissue dissection, including division of the floor of mouth musculature and tongue retraction to access the oropharynx. Panel (c) shows the completed exposure of the C2-C3 junction through a vertical midline pharyngotomy. Panels (d) and (e) depict the layered closure and reapproximation of the mandibular osteotomy with rigid fixation, resulting in preservation of mandibular alignment and satisfactory wound healing on follow-up.

**Figure 3:**
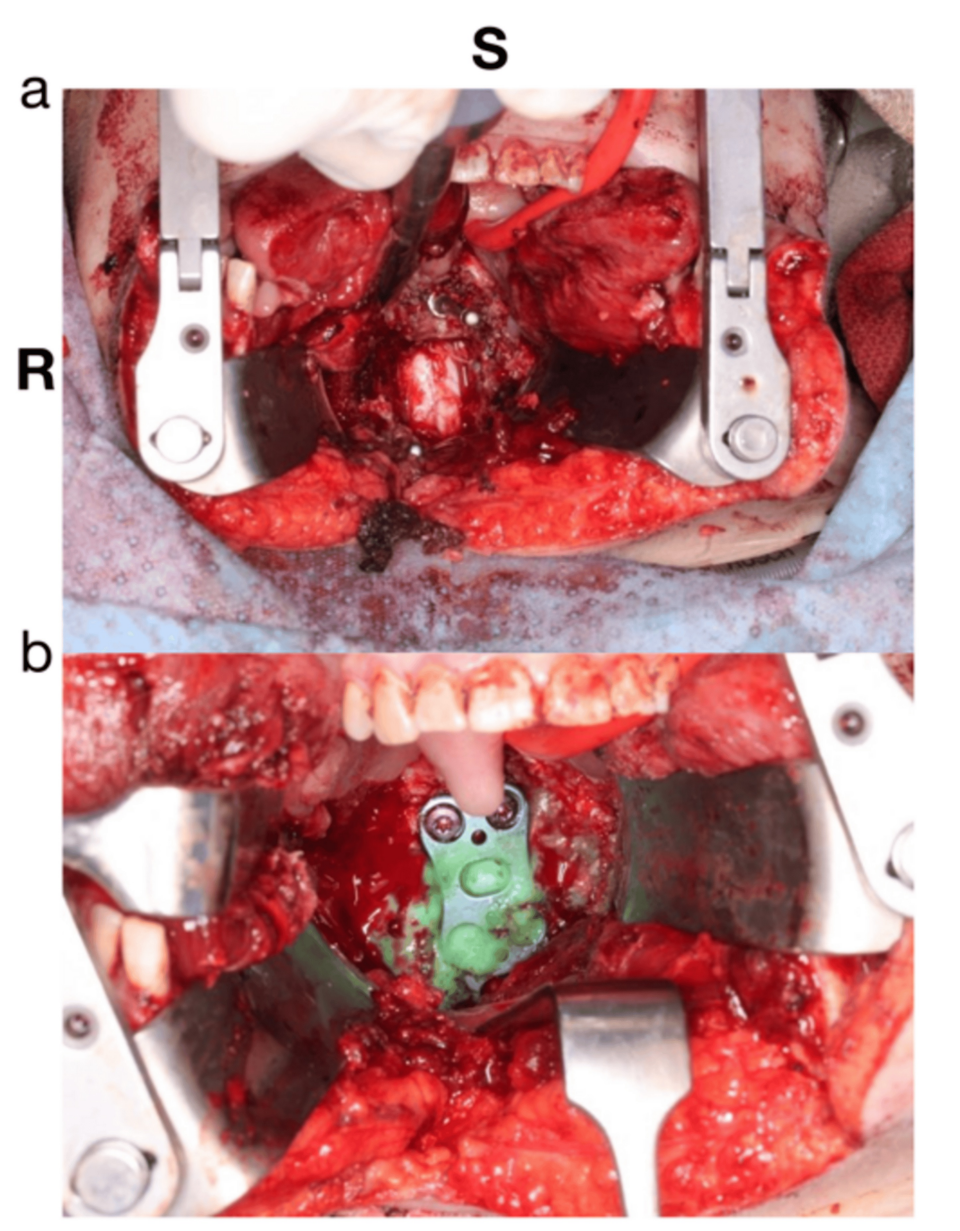
Intraoperative views showing the transmandibular transoral approach for ventral decompression at the C2-C3. (a) Exposure following midline mandibulotomy and glossotomy. The tongue and surrounding soft tissues have been mobilized and retracted using self-retaining retractors, allowing visualization of the anterior vertebral bodies at the C2-C3 level. Prominent venous bleeding was encountered and controlled with bipolar cautery. (b) After complete decompression, a ventral cervical plate is placed and secured to restore anterior column stability. The plate spans the C2-C3 region, and the surrounding field is irrigated, and hemostasis is achieved prior to multilayer closure of the oropharyngeal mucosa.

The key to this approach is the division of the mandible, as well as the soft tissues of the floor of the oral cavity and the tongue. Once these were divided, the oropharynx was opened vertically, and the C2-3 junction was identified and confirmed with fluoroscopy. The intimate attachment of the posterior aspect of the vertebral body of C3 and the thickened ossified posterior longitudinal ligament made the corpectomy especially challenging. Once the layers were egg-shelled using a high-speed diamond drill, a combined elevation and resection technique was used to remove the posterior longitudinal ligament from the spinal cord with fine micro-curettes and microsurgical dissectors, with frequent motor evoked potentials (MEP) signal checks to avoid excessive retraction and manipulation of the spinal cord (Figures 2, 3).

Postoperative Care

Postoperatively, the patient was admitted to the intensive care unit (ICU) and required ventilatory assistance. A lumbar drain was placed for CSF leak management, along with a prolonged prophylactic antibiotic regimen to prevent meningitis. Her neurological exam remained stable, and she was discharged to rehab after 14 days when she was no longer ventilator-dependent and there was no concern for ongoing CSF leak.

Follow-Up

At her six-month follow-up, she could ambulate without assistance, tolerate solid food, and verbally communicate her history in the clinic, with no decline in neurological function.

Case 2: Anterior spinal artery aneurysm (standard transoral approach)

**Figure 4:**
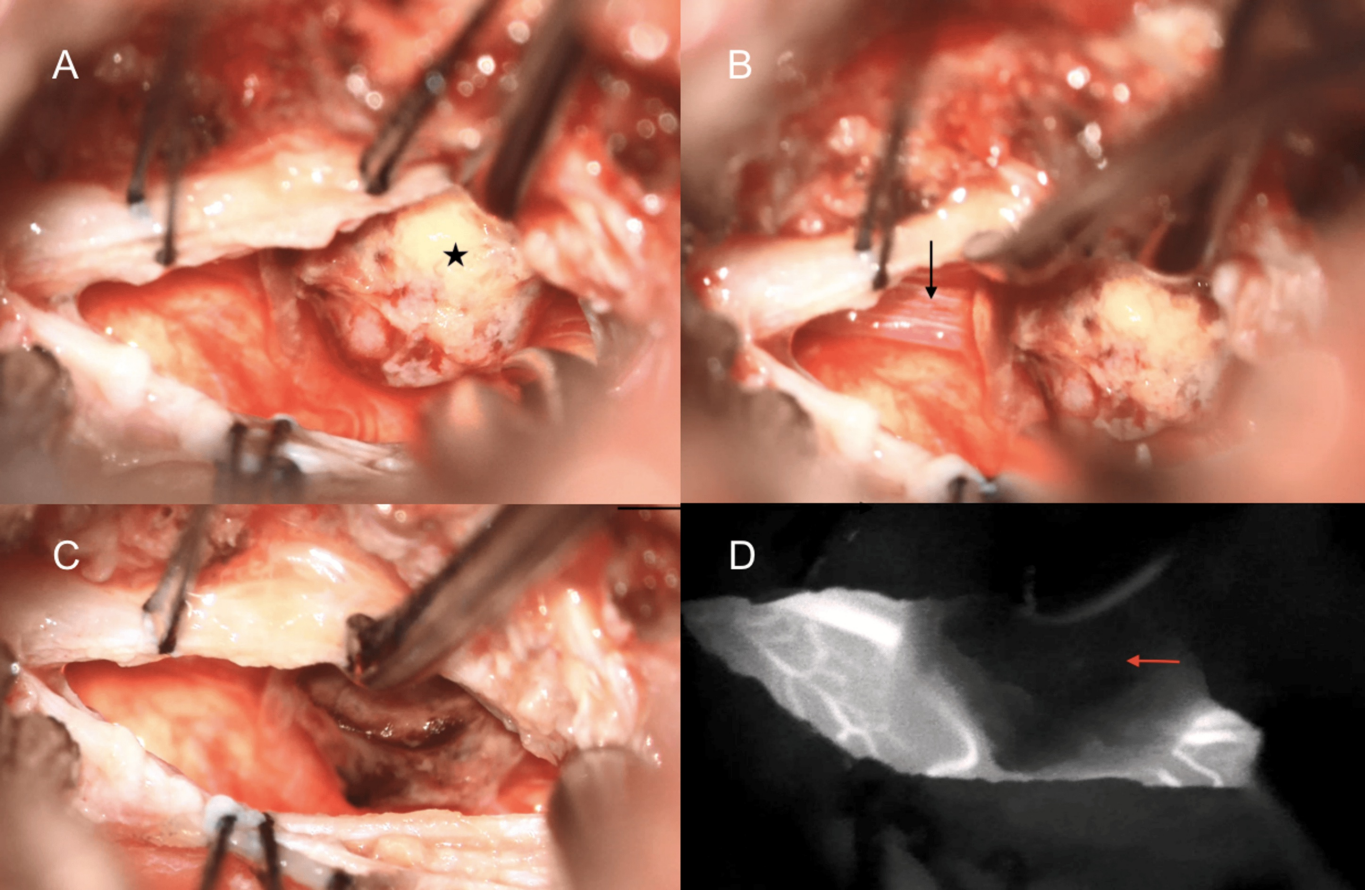
(A) Star: aneurysm. (B) Arrow: ant spinal artery. (C:) Post-aneurysm clipping or cauterization. (D) Intraoperative fluoroscopy showing the drilled C1-C2 decompression.

This was a 75-year-old woman who presented with a sudden onset of headache, nausea, and vomiting and was found to have diffuse subarachnoid hemorrhage and hydrocephalus requiring external ventricular drain insertion. Initial CT angiography and diagnostic cerebral angiogram were negative for identifying the source of the hemorrhage. A repeat angiogram obtained seven days later demonstrated a newly apparent anterior spinal artery aneurysm, suggesting delayed aneurysm opacification or evolution. Given the aneurysm’s ventral cervicomedullary location and small parent vessel caliber, it was not amenable to endovascular intervention. Therefore, a TOA was planned for direct microsurgical obliteration of the aneurysm, as illustrated in Figure 4.

Operation

**Figure 5:**
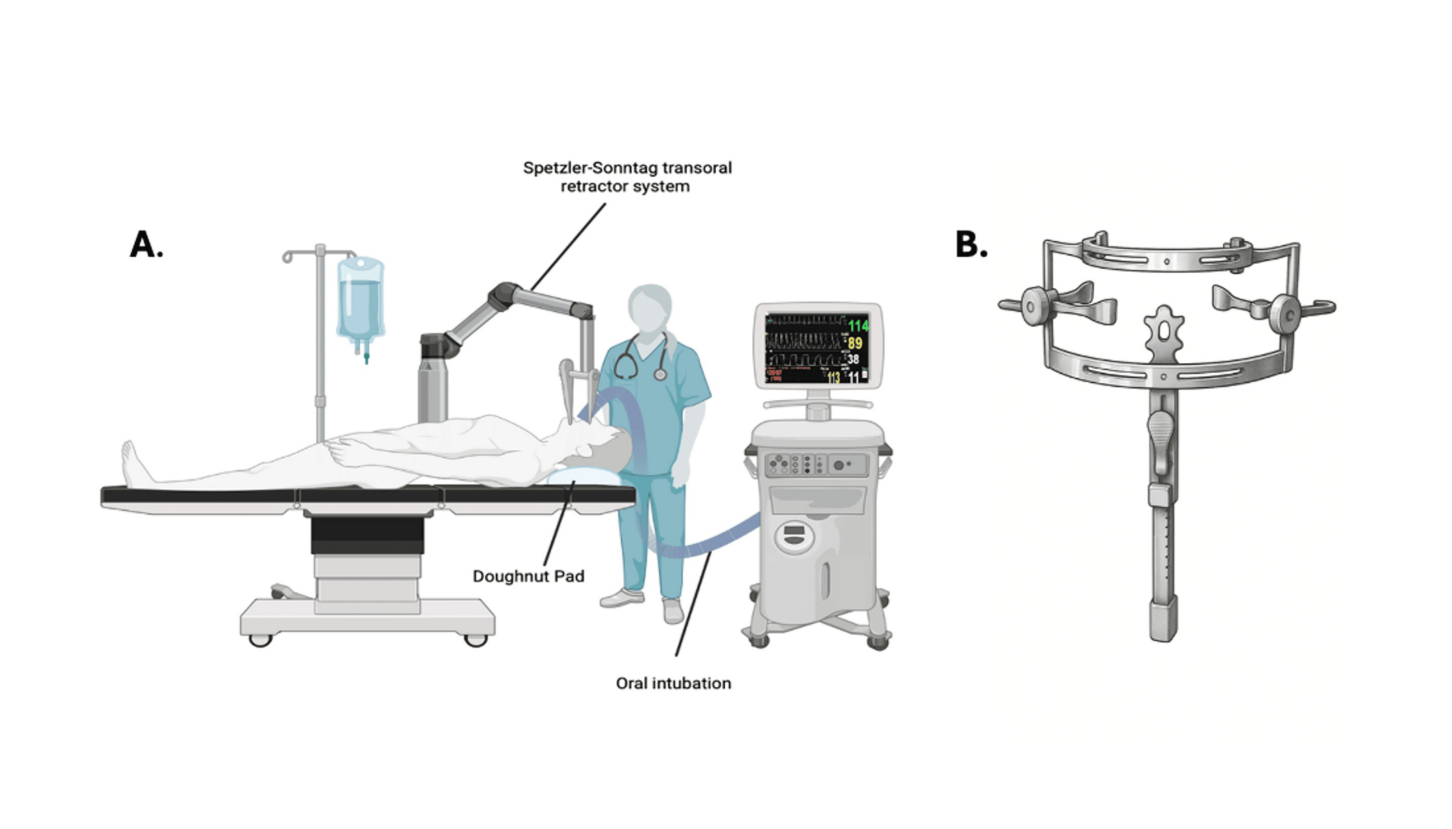
Operative setup for a transoral approach. (A) shows the patient in a supine position with oral intubation and head supported on a doughnut pad, with the Spetzler-Sonntag retractor in place. (B) displays a close-up of the transoral retractor system used to maintain surgical exposure to the craniovertebral junction.

**Figure 6:**
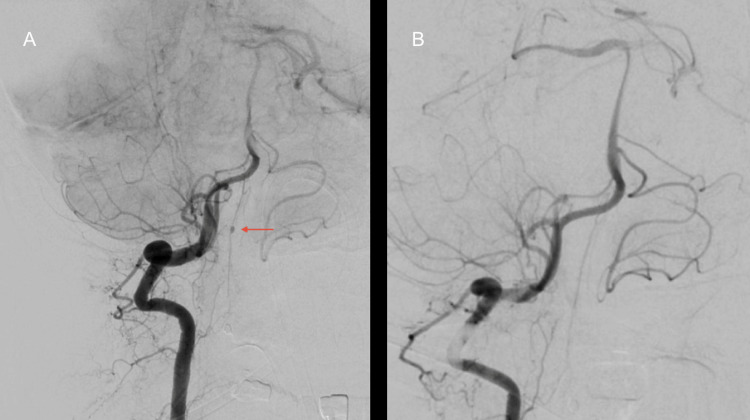
(A) The red arrow shows an aneurysm along the anterior spinal artery at the craniovertebral junction. (B) Postoperative day seven, showing an obliterated aneurysm post clipping.

For this approach, the patient was positioned supine with the head extended and rested on a donut-shaped gel (Figure 5). The oral cavity was held open using a transoral retractor, and the uvula was retracted with sutures. The oropharynx was opened vertically in the midline to access the cervicomedullary junction; this necessitated the drilling of the anterior arch of C1 and the superior aspect of the dense (Figure 6). This allowed us to visualize the dura at the caudal medulla and the initial segment of the spinal cord (C1). The dura was opened at this level, the anterior spinal artery was identified and followed cranially, and the aneurysm was visible as shown in Figure 4. The aneurysm was then obliterated and ligated with the use of bipolar electrocautery and microscissors. The dural closure was done with NUROLON sutures and reinforced with DuraGen and Adherus sealant.

Postoperative Care

The patient remained stable postoperatively but eventually required placement of a gastric feeding tube and a ventriculoperitoneal shunt. She also underwent C1-C6 posterior spinal fusion to address iatrogenic instability resulting from odontoid drilling and disruption of the transverse ligament.

Follow-Up

At the six-month follow-up, the patient was able to tolerate a soft diet with sips, ambulate with assistance, and showed no progression of her neurological deficits.

Case 3: Ventral cervicomedullary pial arteriovenous malformation (transoral-transpalatal approach + Le Fort I osteotomy)

**Figure 7:**
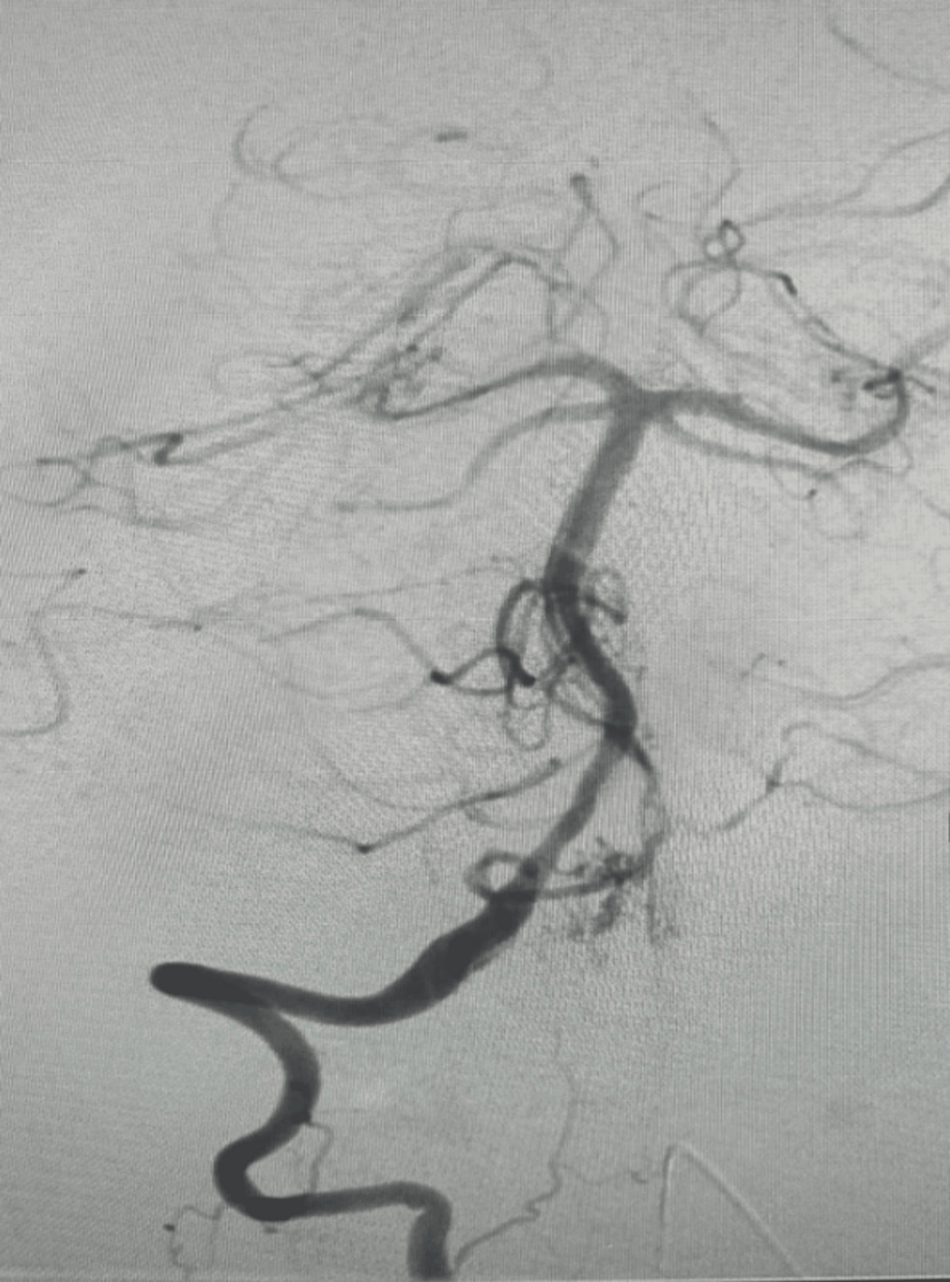
A pial arteriovenous malformation (AVM) located at the ventral cervicomedullary junction.

A 67-year-old woman presented with acute subarachnoid hemorrhage and hydrocephalus. An external ventricular drain (EVD) was placed for increased intracranial pressure management. Cerebral angiography revealed a pial arteriovenous malformation (AVM) located at the ventral cervicomedullary junction, supplied by the right anterior spinal artery (Figure 7). Given the anterior midline location of the lesion, a direct TOA was selected to facilitate ventral exposure and precise microsurgical resection. While an endoscopic endonasal approach (EEA) can provide access to upper clival and ventral brainstem lesions, it offers limited inferior reach and restricts instrument maneuverability for vascular dissection at the lower clival and cervicomedullary junction levels. The TOA allowed a more favorable trajectory to the lower clivus and anterior foramen magnum, enabling bimanual microsurgical control. To achieve adequate rostral exposure, a two-piece Le Fort I osteotomy was performed in collaboration with our oral and maxillofacial surgery (OMFS) colleagues.

Operation

**Figure 8:**
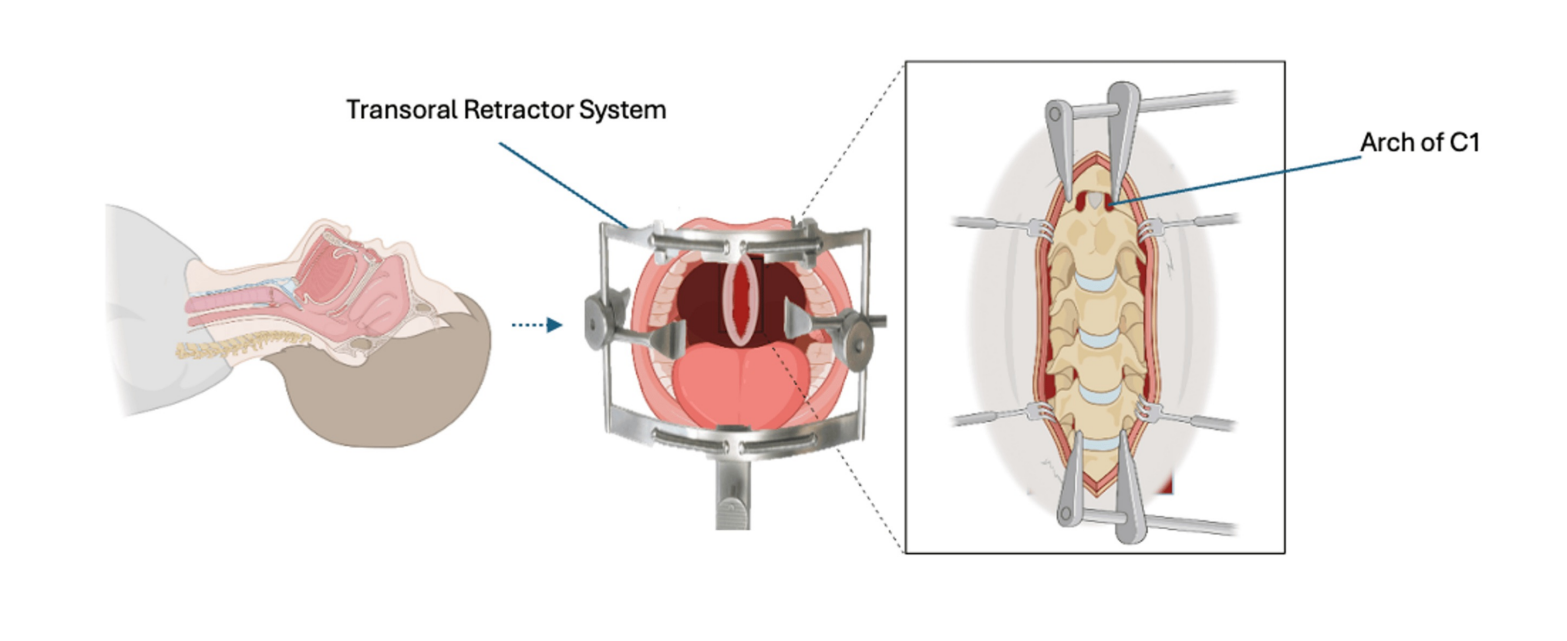
Illustration of the surgical corridor following transpalatal incision. The palate is split to allow direct midline access to the clivus, anterior arch of C1, and superior odontoid. The approach offers an unobstructed trajectory through the oropharynx toward the ventral cervicomedullary junction, minimizing neural retraction while maximizing exposure for microsurgical resection.

**Figure 9:**
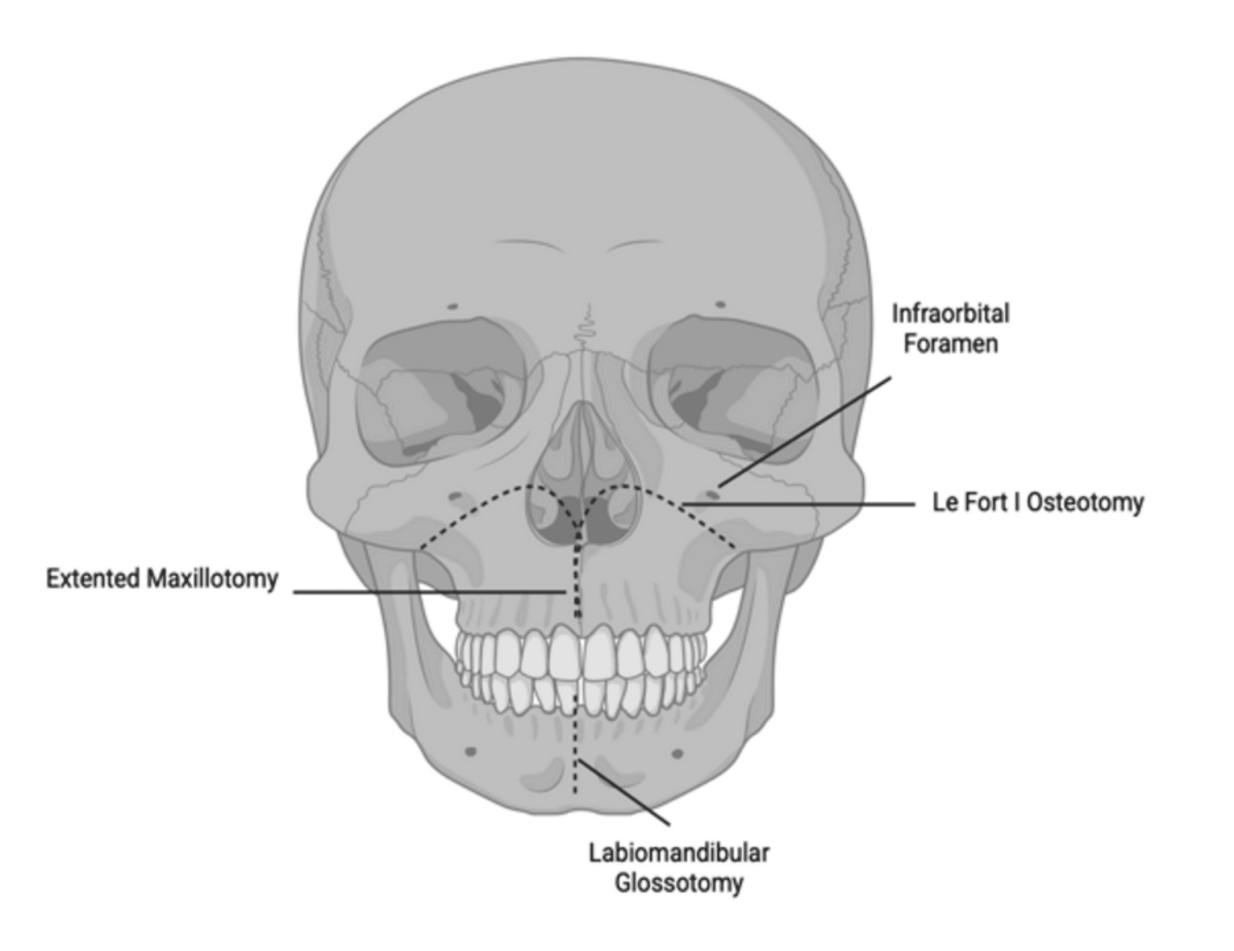
Illustration showing the Le Fort I osteotomy performed to mobilize the maxilla. This maneuver extends superior exposure beyond the oropharyngeal corridor, providing a direct and linear approach to the clivus, anterior arch of C1, and odontoid process during ventral brainstem surgery.

For the approach, the patient was positioned supine with the head fixed in Mayfield pins and the neck in a neutral position to allow intraoperative neuronavigation and maintain craniocervical stability. The oral cavity was prepped and draped in a sterile fashion. The transoral exposure was performed via a vertical midline pharyngeal incision, which was extended following Le Fort I osteotomy and paramedian palatal split (Figure 8). This maneuver enabled direct, linear access to the clivus, C1, and C2 (Figure 9). Under microscopy, the anterior arch of C1 and the superior odontoid were drilled to access the ventral dura. This midline transoral-transpalatal corridor provided a direct and unobstructed path to the lesion, minimizing retraction on neural structures.

After durotomy, the AVM nidus and its feeder from the anterior spinal artery were visualized and circumferentially dissected. The feeding artery was clipped and coagulated, and complete nidus devascularization was confirmed with intraoperative indocyanine green angiography. The nidus was resected en bloc. Dural closure was performed primarily and reinforced with dural substitutes and sealant. The transoral corridor was then closed in a multilayered fashion, and the maxillary osteotomies were reduced and secured using miniplates. Throughout the procedure, neurophysiological monitoring remained stable with no intraoperative changes.

Postoperative Care

In the postoperative period, the patient developed a CSF leak into the oral cavity. She underwent revision transoral exploration and dural repair using an autologous fat graft and sealant reinforcement, accompanied by lumbar drainage. The ventriculoperitoneal shunt was explanted due to persistent egress concerns, and an EVD was placed. The CSF leak was resolved without further complications. To address craniocervical instability resulting from odontoidectomy and C1 arch resection, the patient subsequently underwent C1-C6 posterior instrumentation and fusion.

Follow-Up

At the six-month follow-up, she remained neurologically stable and functionally independent, without recurrent CSF leakage or hydrocephalus.

Table 1 provides a summary of illustrative cases.

**Table 1:** Summary of illustrative cases. AVM: arteriovenous malformation; CSF: cerebrospinal fluid; VP: ventriculoperitoneal.

Case	Age/sex	Diagnosis	Surgical approach	Procedure	Complications	Outcome
1	38F	Klippel-Feil syndrome with progressive myelopathy	Transmandibular transoral	C3 corpectomy and decompression	CSF leak, tracheostomy	Independent ambulation, no neurologic decline at 6-month follow-up
2	75F	Anterior spinal artery aneurysm	Standard transoral	Odontoid drilling, aneurysm clipping	VP shunt placement, prolonged dysphagia	Ambulates with assistance, neurologically stable at 6 months
3	67F	Ventral cervicomedullary AVM	Transoral-transpalatal with Le Fort I osteotomy	Microsurgical AVM resection	CSF leak requiring revision, posterior C1–C6 fusion	Independent, no CSF leak or hydrocephalus at 6-month follow-up

## Discussion

The TOA has long been regarded as the gold standard for accessing ventral CVJ pathologies, especially in patients with irreducible lesions compressing the brainstem and upper spinal cord. Early use of TOA demonstrated favorable decompression outcomes but with notable complications. Building upon this historical foundation, our case series demonstrates the continued relevance and adaptability of the TOA in the modern microsurgical era, specifically for highly selected, non-degenerative vascular and congenital CVJ pathologies where direct ventral access remains indispensable. In one of the earliest and most influential studies, Menezes and VanGilder (1988) reported significant neurological improvement in 72 patients with rheumatoid arthritis, basilar invagination, and AAD, though most required posterior fusion due to resultant instability [20]. Similarly, Di Lorenzo (1992) highlighted the effectiveness of TOA in decompression but raised concerns over frequent postoperative instability [21].

Hadley et al. (1989) treated more than 50 patients with extradural compression and observed good surgical outcomes but noted complications such as dysphagia and CSF leaks. These early experiences established TOA as a powerful yet high-risk option for patients with midline ventral lesions [22].

To reduce complications, endoscopic techniques were gradually introduced. Frempong-Boadu et al. (2002) utilized endoscopic assistance in seven patients, reporting excellent decompression outcomes, though some CSF leaks still occurred [23]. Building on this, Husain et al. (2006) and Yadav et al. (2013) explored fully endoscopic transoral odontoidectomy in irreducible AAD, achieving 90-91% clinical improvement rates. However, transient swallowing issues and occasional infections persisted, underscoring that less invasive techniques still present technical and postoperative challenges [24,25].

As newer techniques such as the EEA emerged, comparisons with TOA became more frequent. A 2016 meta-analysis by Shriver et al. pooled 282 cases and found that both approaches achieved similar neurological outcomes, but EEA offered better postoperative recovery, fewer tracheostomies, faster resumption of oral intake, and lower dysphagia rates [26]. Heller et al. (2021) supported these findings, particularly in non-neoplastic cases. However, EEA has limitations in accessing lower CVJ lesions and remains more technically demanding [27].

Other strategies have been explored for specific scenarios. Doglietto et al. (2019) proposed combining TOA with EEA to enhance exposure to the upper cervical spine in cases with challenging anatomy [28]. For ventrolateral lesions, Kawashima et al. (2019) and Sen et al. (2005) demonstrated the utility of far lateral and transcondylar approaches, respectively, which offer wider exposure but often necessitate occipitocervical fusion (OCF) [29,30].

Case reports continue to underscore TOA’s utility in select scenarios. Haq et al. (2024) used a two-stage TOA and posterior fusion for an odontoid fracture [31]. Jeszenszky et al. (2018) applied a closed transoral reduction in pediatric atlantoaxial rotatory subluxation (AARS) [32]. These reports highlight that despite the rise of minimally invasive and lateral alternatives, TOA remains a valuable technique in anatomically constrained cases.

Complications such as CSF leakage and dysphagia remain significant concerns following TOA. Even with improved closure techniques and intraoperative strategies, these risks persist. When CSF leaks occur in the open oropharyngeal setting, the risk of infection, including meningitis, increases. Several authors have recommended using fat grafts, sealants, and even prophylactic lumbar drainage in high-risk patients [2-10].

Despite its invasiveness, TOA remains a valuable technique when applied selectively, particularly for midline, ventrally located lesions involving the axis (C2 vertebra) or below. With favorable anatomy and thorough surgical planning, TOA can achieve excellent decompression and meaningful neurological recovery. However, its use should be reserved for cases where its specific advantages outweigh the associated risks.

Future directions

Future advances should focus on improving the safety and efficacy of the TOA while minimizing complications. Intraoperative navigation, robotics, advanced imaging, and improved closure techniques can reduce risks such as CSF leakage and infection. Innovations in dural reinforcement and vascular protection may further enhance outcomes. Multi-institutional registries and knowledge sharing will help track rare CVJ pathologies and guide clinical decision-making. Preserving TOA training through simulation, case-based teaching, and collaboration with maxillofacial teams is essential to maintain surgical proficiency as newer approaches gain popularity.

## Conclusions

The TOA remains a vital surgical option for managing ventral CVJ pathologies. Although less invasive alternatives such as the EEA and posterior approaches have demonstrated promise, they may not provide sufficient exposure for deep-seated or midline lesions. Our case series highlights the enduring relevance of TOA in rare and complex scenarios where other approaches are limited. When applied with meticulous preoperative planning and technical precision, TOA continues to offer reliable decompression and meaningful neurological stabilization. In carefully selected patients with ventral pathology inaccessible by other approaches, TOA provides a definitive surgical corridor, requiring disciplined execution, multidisciplinary coordination, and vigilance in managing approach-related complications.
